# On Fracture of the Skull in Children

**Published:** 1846-11

**Authors:** F. H. Hamilton

**Affiliations:** Buffalo


### ART. IV.—*On Fracture of the Skull in Children.* By F. H. Hamilton, M. D.

In relation to the propriety, or necessity of an operation in cases of fracture of the skull, occurring in infancy or early childhood, we have, I believe, been generally silent. We speak always of fractures of the skull as they exist in adults; but the rules of practice applicable in these cases are seldom applicable to children, and the surgeon who does not recognise peculiarities in the cranial anatomy of the latter, must frequently, I think, commit very gross blunders. Thus,

Until the second or third year of life, and occasionaly much later, the fontanelles are open.

During the same period, also, very little hard matter has been deposited in the structure of the bones composing the cranium; they are soft and easily bent, and may with little force be depressed, and with as little force replaced. This resilience of the skull gradually diminishes as life advanees, but is generally considerable even at the fifth or eighth year.

In early life the cranial bones are very thin, and not until about the fifth year can a distinct diploic structure be detected. The dura mater is also very firmly adherent to the skull.

From the two circumstances first mentioned, viz. the open state of the fontanelles, and the pliancy of the bones, it happens that if by accident the skull is forced in upon the brain during early life, unless the depression is very considerable, no coma is produced; the brain pressing out at the fontanelles, or displacing the bones upon the opposite side, to a sufficient extent to prevent compression. From the softness of the bones it also happens that the skull is rather *bent* in than broken, and no sharp spiculae exist which can penetrate and disturb the brain.

While their elasticity is such, that generally after a time, longer or shorter, according to the age of the patient, and depth of the depression, they resume spontaneously their original position.

*Treatment.* Finally, from all these facts we infer,

1. That if a fracture with depression occurs in a child, and no signs of compression follow, no operation is justifiable. The depression is harmless and the probability is that in a few weeks or months the deformity will disappear.

2. That if symptoms of compression do actually exist, and there is no external wound which will at once admit the elevator to the fracture, unless the coma has continued several hours, no operation, not even the incision of the scalp is generally proper. And if, after the lapse of "several hours" the coma continues, yet is not great, or if it is gradually diminishing, it is still well to delay an operation.

3. If in connection with the coma there exists also a considerable external wound, so that instruments can at once be applied to the skull, and the elevator has been used with skill and without effect, still the propriety of resorting to the saw or trephine may be doubtful. Because, at this period of life it is difficult to make a successful operation. The trephine must traverse the entire thickness of the skull, (not *nearly* through, or only through *at points*, leaving the balance of the inner plate to be *broken up* with the levator as we practice in adults, since the bones are too soft to be broken up by the levator;) and then the teeth of the saw are almost certain to lacerate the dura mater, and the more so because this membrane is, at this age more closely attached, and the mere concussive effects of the blow does never (as often happens in *adults*) separate it from the bone. Owing to this closer union also of the dura matter to the skull, the separation of a fragment of the latter is accomplished with greater violence and produces more inflammation. Having made a sufficient opening with the trephine, to admit the elevator, the depressed fragments are not always easily elevated, owing to the bending of the edge under which the elevator rests, so that often it becomes necessary to introduce the levator again at another point: and still farther to increase the difficulty, the depression is generally much more extensive in children, than in adults.

When we regard this accumulation of difficulties, and place against it the fact that the bones do even in some of the most desperate looking cases come back spontaneously, we shall conclude that *an operation can seldom be necessary or proper*. The following cases may serve to illustrate these positions.

*Case* 1. *Depression but no coma, bones resumed their places spontaneously.*—Nov. 20th, 1844. Infant son of James McGraith, Rochester, aged 18 months, fell from a pair of stairs six feet, the head striking upon a stone. Right parietal bone was depressed half an inch, and for about two inches in length; scalp torn up at point of fracture. One hour after the accident I saw the child; he was nursing, no signs of cerebral disturbance; seemed playful and free from pain. I applied cool lotions and directed a mild cathartic. Three weeks after, while absent, I learned from another physician that the bones had nearly resumed their places, and that the child had never seemed to suffer from the accident.

*Case* 2. *Depression without coma, and bones gradually resuming their position.*—Sept. 26, 1846. Dr. Wilson, the intelligent native physician of Cattaraugus, brought to me his son aged five months. Two weeks before, he was thrown with violence from a buggy upon the ground while descending a hill, and the right parietal bone was forced in three quarters of an inch, and over a space covering two and a half inches in length by one and a half in breadth. The child has never been comatose. During the two first days he was easily startled, but was otherwise well. When I saw him on the 26th the indentation had diminished in depth a little; he had not suffered in any way from the accident except as stated on the two first days. He was entirely well. Dr. Wilson had been advised by physicians to make an operation.

*Case* 3. *Fracture and depression, with extensive suppuration under skin of scalp, but no coma.*—Aug. 10, 1835. Jane Corey, aged 3 years, Cayuga Co.; was climbing through a rail fence, when a rail fell and struck her near the top of the head. I saw her two weeks after the accident, and found a large abscess under scalp which I opened. The skull was broken and depressed considerably, but she had never had coma, or any sign of cerebral disturbance. I did not elevate the bones. A few months later she was well.

*Case* 4. *Fracture and extensive depression, with slight external wound; no coma; bones restored spontaneously.*—Jan 2, 1840. While at Fairfield, Herkimer Co., I was called in the night to the town of Newport; the physician who sent for me stating in his note that the "whole side of the child's head" was "driven in" and that my instruments would be indispensable. The parents had been riding in a sleigh, the horse became frightened and unmanageable, and dashing finally against an awning post, both parents and child were thrown with great violence upon the side walk, the head of the child striking a corner of the curb stone.

I found an infant aged about 14 months; one side of the skull fractured and driven in to the depth at the deepest point of nearly an inch, and the depression covering about four inches by two and a half; with a slight wound over the fracture. When first taken up, the child was pale and apparently insensible, but it soon recovered, and now, four hours after the accident, he was bright and intelligent—he had slept and nursed. A cathartic and cooling lotions were recommended. About three weeks after, at my special request, the parents brought the child before the class, and I was happy to show them that the little fellow was not only quite well, but that the skull had again resumed its place, and we need have no further solicitude about "cautiousness" and half a dozen other important organs, whose house-room had been so seriously curtailed.

*Case* 5. *Depression and fracture; extensive; deep and continued coma; effusion of serum, eventual recovery and spontaneous replacement of bones.*—1837. Son of P. Parker of Scipio, aged 3 years, was playing in the barn, when a scantling fell from the scaffold and one end struck him obliquely on the left parietal bone, crushing in the bones and apparently extinguishing life for a time. When I saw him about four hours after the accident he was in a deep coma, pupils dilated and immoveable, eyes crossed, pulse slow and very feeble, breathing labored. The whole length of the parietal bone was broken in two parallel lines, intersected in the center by a cross fracture, at which central point the depression was very great. After some delay, Dr. Gilmore the atttending physician, and myself, determined to advise the parents that an operation would be necessary, and that we would proceed immediately. We proposed first to make an incision and try the elevator, and in case of failure we had determined to trephine. The parents refused peremptorily to have *any* operation made. They believed the child would die, and to injure him farther seemed to them barbarous. We were forced to submit. We remained during the night and before morning we bled him and administered by injection, oil and turpentine.

*Next morning.*—"Injection has operated; coma less profound; can arouse him slightly;" continued injections, cold lotions to head, friction to extremeties, &c., &c.

*Third day.*—"Has improved considerably; wakes easily; slight squinting; pupil less dilated; can swallow with some effort; bones less depressed; right arm and leg completely paralysed."

*Twelfth day.*—"Still improving; paralysis gone; squints some. Before the injury he had learned to talk very intelligibly. He now begins to say "Pa," and "Ma," but seems to have forgotten all other language."

About the *third week*, a small fluctuating tumor made its appearance over the anterior portion of the fracture, which on careful examination we diagnosed as an effusion of serum under the scalp and communicating with the meninges of the brain. It disappeared gradually after several weeks, and the bones having resumed entirely their original position, the cure was finally complete.

I still believe that in this case an operation would have been *proper*, yet the result proves that it was not *necessary*.

*Buffalo, October*, 1846.

